# Seleno-Metabolites and Their Precursors: A New Dawn for Several Illnesses?

**DOI:** 10.3390/metabo12090874

**Published:** 2022-09-16

**Authors:** Cristina Morán-Serradilla, Eduardo Angulo-Elizari, Andreina Henriquez-Figuereo, Carmen Sanmartín, Arun K. Sharma, Daniel Plano

**Affiliations:** 1 Department of Pharmaceutical Technology and Chemistry, University of Navarra, Irunlarrea 1, E-31008 Pamplona, Spain; 2 Department of Pharmacology, Penn State College of Medicine, 500 University Drive, Hershey, PA 17033, USA; 3 Penn State Cancer Institute, 500 University Drive, Hershey, PA 17033, USA

**Keywords:** selenium, seleno-metabolites, H_2_Se-precursors, CH_3_SeH-precursors, bioactive agents, cancer, cardiovascular diseases, neurodegenerative diseases

## Abstract

Selenium (Se) is an essential element for human health as it is involved in different physiological functions. Moreover, a great number of Se compounds can be considered potential agents in the prevention and treatment of some diseases. It is widely recognized that Se activity is related to multiple factors, such as its chemical form, dose, and its metabolism. The understanding of its complex biochemistry is necessary as it has been demonstrated that the metabolites of the Se molecules used to be the ones that exert the biological activity. Therefore, the aim of this review is to summarize the recent information about its most remarkable metabolites of acknowledged biological effects: hydrogen selenide (HSe^−^/H_2_Se) and methylselenol (CH_3_SeH). In addition, special attention is paid to the main seleno-containing precursors of these derivatives and their role in different pathologies.

## 1. Introduction

**Figure 1:**
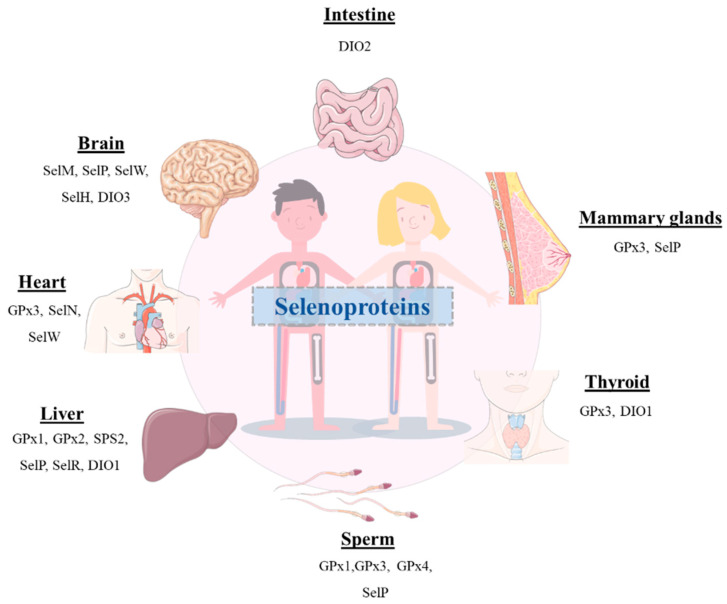
Schematic representation of the presence of several selenoproteins in different organs of the human body.

Although selenium (Se) was discovered in 1817 by the Swedish chemist and doctor Jacob Berzelius, the importance of this multifunctional trace element was not recognized until many years after [1]. This was a consequence of the prejudices related to the fact that Se was considered a harmful toxic substance. Thus, the research in the field devoted to obtaining deeper insight into the therapeutic potential use of this element was carried out by a small group of scientists worldwide [2]. In 1957, Klaus Schwartz and Calvin Foltz demonstrated its benefits, as it could prevent liver necrosis in vitamin-E deficient rats [3]. Since then, an enormous effort has been directed to ascertain the crucial role of this element in the human body. Nevertheless, it should be emphasized that either deficient or elevated levels of Se can result in harmful outcomes [4,5]. Given its narrow therapeutic window, a concentration limit has been established for Se to promote its beneficial effects and prevent its toxicity. Currently, the daily allowance of Se intake recommended by the World Health Organization (WHO) is set at 55 μg for adults, and the main dietary sources of this element include grains, seafood, meat, vegetables, and nuts [1,3,6,7,8,9]. Furthermore, it has been well-documented that Se is a pivotal component of selenoproteins that are involved in a large number of bioactivities exerted by essential enzymes (Figure 1). Therefore, it can play a key role in the prevention and treatment of several pathologies [10,11,12,13,14,15,16,17,18,19,20,21].

**Figure 2:**
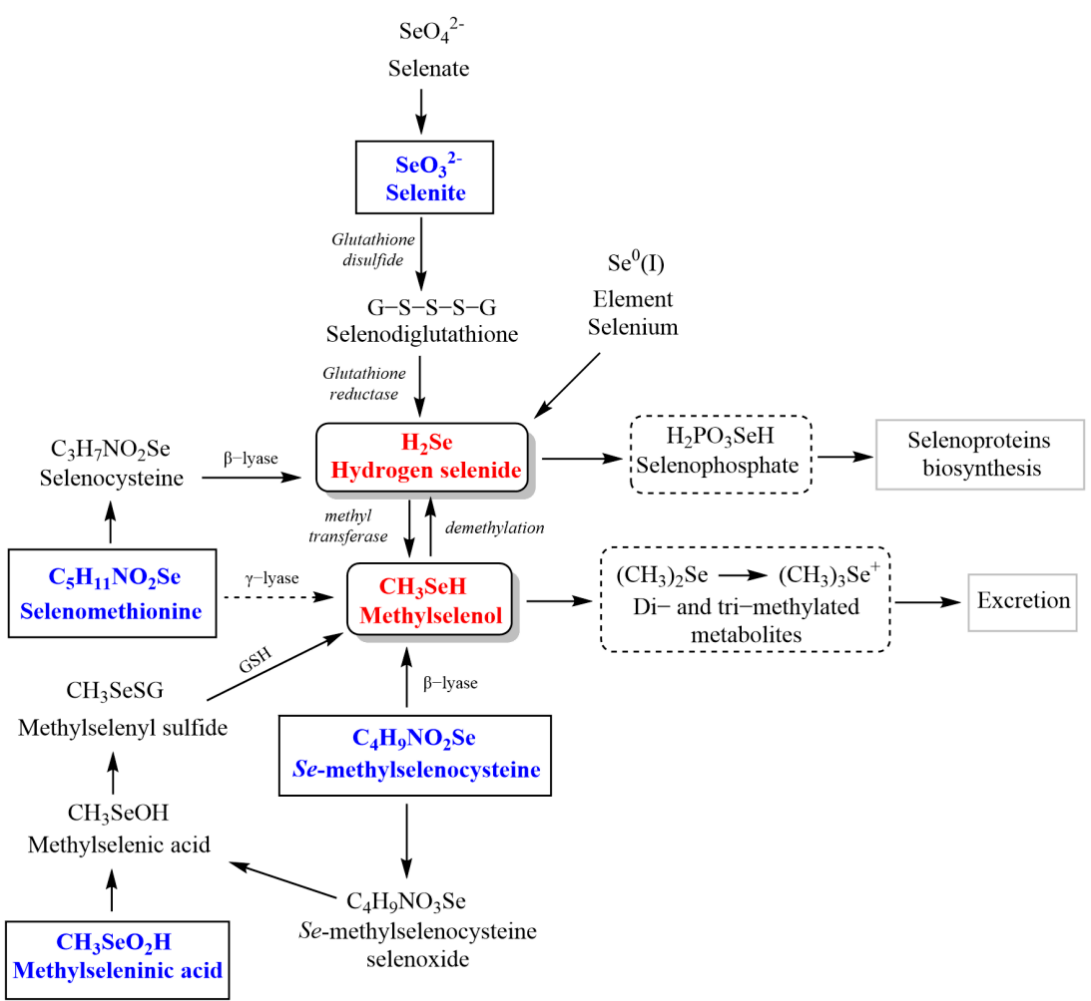
Metabolic pathways of some Se-containing compounds that are the precursors of H_2_Se and CH_3_SeH.

Notably, it has been reported that the metabolism of selenocompounds is complex and closely regulated (Figure 2). Hydrogen selenide (HSe^−^/H_2_Se) and methylselenol (CH_3_SeH) are the main crucial metabolites involved in the biological properties of these molecules [22]. Hydrogen selenide can be generated from organic and inorganic selenocompounds (i.e., selenite, element Se and *L*-selenocysteine). It is involved in several reactions in cells and can be reversely methylated to give CH_3_SeH and later to dimethylselenide ((CH_3_)_2_Se) [23,24,25]. Additionally, it is required for the synthesis of selenocysteine (SeCys), which is incorporated into selenoproteins. This metabolite has also a significant role in many biological functions, such as Se homeostasis, the correct function of the thyroid, and several endogenous antioxidant systems [26,27,28,29,30,31,32,33]. It can produce reactive oxygen species (ROS) which can lead to cell cycle arrest by blocking the S/G2 phase, single-strand DNA lesions or nicks, and apoptosis, as reviewed in [34]. Noteworthy, hydrogen selenide shares some features with the gasotransmitters (NO, CO, and H_2_S) and has been recently proposed as the fourth member of the endogenous gasotransmitters [25,26,28,29]. On the other hand, CH_3_SeH plays an important role in the innate immune response as it can activate natural killer (NK) cells and raise the expression of natural killer group 2 member D (NKG2D) ligands and interferon (IFN) [22,24,35]. Moreover, a large body of existing literature indicates that CH_3_SeH can exert anticancer activity [34,36,37,38,39]. Over the last two decades, convincing evidence has been accumulated indicating that its underlying mechanism may be attributed to increasing ROS formation, triggering apoptosis, inducing DNA damage, and inhibiting angiogenesis [40]. In addition, CH_3_SeH can behave as a protein redox modulator by targeting the cysteine residues [38]. Nonetheless, regarding its elevated reactivity and volatility, antitumoral therapy would require its production in situ or the use of precursors, such as methylseleninic acid, *Se*-methylselenocysteine, and selenomethionine [38,39].

In this regard, the main purpose of this review is to present a survey of the literature that demonstrates the therapeutical application of some of the most remarkable H_2_Se and CH_3_SeH precursors. These insights may aid future investigations aimed at discovering and improving treatments against a wide range of pathologies.

## 2. Hydrogen Selenide Precursors

### 2.1. Sodium Selenite

Emerging evidence suggests that selenium-containing molecules play an important role in a great number of pathologies. A plethora of research has been dedicated towards selenites (Se^4+^) as they ascertain a crucial role in medicine. They can undergo oxidation and reduction reactions as they can be reduced to their divalent cation (Se^2+^) that can act as an oxidant [5,41]. Amidst these inorganic Se compounds, sodium selenite (Na_2_SeO_3_, SS) has attracted considerable attention in the scientific community, and it is present in many dietary supplements. Its short-term toxicity can be considered minimal and has been well-described [42]. The metabolism of this compound to form hydrogen selenide (H_2_Se, the key metabolite of Se in cells) has been thoroughly studied over the past decades [24]. In the following subsections, the role of this compound in different diseases will be discussed.

#### 2.1.1. Antibacterial Activity of Sodium Selenite

It is a well-known fact that bacterial multidrug resistance is a major ongoing clinical issue as there is a growing number of strains resistant to the available antimicrobials [43,44,45]. Recent reports have shown that Na_2_SeO_3_ can enhance the sensitivity of different human pathogenic bacteria against traditional antibiotics: *Staphylococcus aureus* (*S. aureus*) ATCC 25923 to oxacillin, cloxacillin and ampicillin/sulbactam and methicillin-resistant *S. aureus* (MRSA) to neomycin [46,47,48]. Furthermore, it has been proposed as an alternative therapeutic option for *Clostridium difficile* (*C. difficile*) infection (CDI) as it can cause a depletion of the virulence and toxicity of this pathogen by reducing the exotoxin production and the spore outgrowth. Furthermore, significantly increased sensitivity to ciprofloxacin was observed when the samples were treated with the combination of Na_2_SeO_3_ and this antibiotic. In view of the results, Na_2_SeO_3_ can be considered as a potential adjuvant in the treatment of CDI, albeit further studies are required [46]. Furthermore, in vitro and in vivo studies have unveiled that Na_2_SeO_3_ has a bactericidal effect on the gastric pathogen *Helicobacter pylori* (*H. pylori*) and possesses ulcer healing properties. This bacterium has drawn great attention owing to its capacity to cause gastric cancer, peptic ulcer disease, and chronic gastritis [46,48,49]. Keeping in mind the aforesaid antecedents, Na_2_SeO_3_ can be considered a highly favorable candidate for the treatment against a wide spectrum of bacteria.

#### 2.1.2. Sodium Selenite and Asthma

Asthma is one of the most common chronic respiratory diseases and it is characterized by airway hyper-reactivity, lung inflammation, and airway obstruction [50]. Studies have suggested that oxidative stress plays a key role in the etiology of asthma [51,52,53]. Therefore, it has been proposed that dietary antioxidants might limit this phenomenon in the lungs and reduce the symptoms related to this respiratory disorder [51]. For this reason, some studies have been carried out to determine if a higher selenium intake could result in a plausible depletion of the inflammation triggered by asthma [54]. A mouse model of asthma has demonstrated the anti-inflammatory properties of Na_2_SeO_3_. An increase in the cell adhesion proteins expression in the lung and the activation of the nuclear factor *kappa* B (NF-κB) were observed after the injection of this inorganic molecule into the peritoneum of allergen (ovalbumin)-sensitized mice. In addition, the supplementation of the human airway epithelial cells (A549) with Na_2_SeO_3_ raised the activity of the selenium-dependent glutathione peroxidases in the lung tissue. It also inhibited the hydrogen peroxidase formation and the activation of the NF-κB. The data obtained from this study suggest that Na_2_SeO_3_ can regulate the activity of NF-κB by enhancing the activity of the Se-dependent glutathione peroxidase, resulting in the removal of potential activators of this transcriptional factor, and by the direct oxidation of critical sulfhydryl groups present in the NF-κB [24].

#### 2.1.3. Sodium Selenite and Cancer

Cancer remains one of the major serious and most challenging public health problems that afflicts millions of people worldwide. According to the WHO, lung, breast, colorectal, and prostate cancers stand out as the most common ones, whilst lung cancer has the highest mortality rates [55]. At present, although there are some treatments available for this disease, they are usually associated with detrimental side effects, drug resistance, reduced treatment adherence, and a decreased quality of life for the patients [56,57,58,59,60]. There is unquestionably a major unmet need in this regard, thereby a plethora of studies are dedicated to discovering different strategies for treating cancer. Among the wide range of health benefits ascribed to Se, its role in cancer prevention and treatment has drawn considerable attention [5,6,61,62,63,64,65,66,67]. In fact, its effect depends on some factors, such as the chemical form, bioavailability, dose, and cancer type [41,68]. There is mounting evidence that tumor cells, especially highly resistant cancer cells to cytostatic drugs, are more sensitive to Se than normal ones [69,70,71]. Thereby, Se compounds can be considered feasible therapeutic drug candidates for treating cancer [72]. Na_2_SeO_3_ has attracted great research interest in recent years due to its chemopreventive and antitumoral properties at non-toxic doses. It has been found that its supplementation to patients can reduce the side effects of chemotherapy [41]. Moreover, a great number of studies have demonstrated that it exerts an antitumoral activity in a wide range of cancers: cervical, breast, colorectal carcinoma, lymphoma, leukemia, thyroid, bladder, glioblastoma, pancreatic, and liver cancer cell lines [41,68,72,73,74,75,76,77,78,79,80,81].

#### 2.1.4. Sodium Selenite and Cardiovascular Diseases

Cardiovascular diseases (CVD) are the leading cause of mortality worldwide. There is a growing body of evidence that Se is involved in proper cardiovascular function and its deficiency is linked to different CVD, such as atherosclerosis, heart failure, myocardial infarction, and Keshan’s disease [5,7,10,82,83]. The last one is endemic cardiomyopathy which was first reported in 1935 in Keshan County in northeast China. The exact etiology of this disease remains undetermined, although some possible causes have been reported (i.e., viral infection, malnutrition, etc.) [28,82,84,85]. Bearing in mind the likely linkage between the micronutrient Se and cardiovascular activity, a wide variety of studies have been carried out in different settings to ascertain the crucial role of Se-containing compounds in CVD [5,86].

The adaptation of the vascular wall structure to the massive proliferation and reduced apoptosis of the vascular smooth muscle cells (VSMCs) is known as vascular remodeling (VR). It is widely recognized that pathological VR is responsible for numerous cardiovascular conditions, such as atherosclerosis, pulmonary arterial hypertension, and post-angioplasty restenosis [87,88]. Even though stent implantation is an available treatment for the attenuation of the obstruction of the coronary flow related to atherosclerosis pathologies, its detrimental effects have raised the interest in finding new alternatives. An extensive number of literature reports have indicated that Na_2_SeO_3_ presents a wide range of potential therapeutic effects, including an anti-atherosclerotic activity [89,90,91]. It has been demonstrated that it can mitigate vascular endothelial dysfunction as well as vascular inflammation [89]. Furthermore, a recent study has shown that this inorganic compound exerts mediatory effects on the proliferation and apoptosis of vascular smooth muscle cells (VSMCs) in two different vascular injuries in vivo models: rat carotid artery balloon injury and arterial hypertension. Although it is necessary to gain further insight into the underlying mechanism of its activity, the data suggest that it might be mediated by the suppression of the AKT and ERK pathways. In view of the former, Na_2_SeO_3_ can be considered an attractive potential drug candidate for the treatment of VR-related pathologies [87].

#### 2.1.5. Sodium Selenite and Diabetes

Diabetes mellitus (DM) is a chronic metabolic disease. Type 1 DM (T1DM), also known as “insulin-dependent DM”, is an autoimmune disorder that is mainly developed at a young age. It is characterized by progressive destruction of the insulin-producing β-pancreatic cells and the insufficient production of insulin [92,93,94]. Currently, the main treatments available for T1DM consist of insulin injections to keep the blood glucose levels. Nevertheless, it is important to highlight that these can only temporarily lower the levels and are associated with some detrimental complications, such as neuropathy, nephropathy, and retinopathy. Therefore, further research is needed to improve the treatment of this disease. Additionally, it is a well-known fact that diabetic retinopathy can be triggered by numerous factors, such as ROS [92,93,94,95]. For this reason, greater effort has been recently made to obtain deeper insight into the potential use of Se species as antidiabetic agents. Although few data are available, the antidiabetic effect of the Na_2_SeO_3_ has already been investigated. Recent studies have reported that this might be explained by the suppression of blood glucose levels, and it has been proven to reduce diabetic retinopathy in rats. Authors believe that this might be caused by a raise in the level of insulin secretion in the β-pancreatic cells [92]. Given the abovementioned facts, these findings should be considered for developing possible future therapeutic strategies for this disease.

#### 2.1.6. Sodium Selenite and Neurodegenerative Diseases

Oxidative stress and mitochondrial dysfunction are characteristic features of some neurodegenerative pathologies, such as Alzheimer’s disease (AD), Parkinson’s disease (PD), and Huntington’s disease (HD). It is worth mentioning that over the past years the potential application of antioxidants in the treatment of these diseases has been explored [96,97]. In this context, the role of Na_2_SeO_3_ in the oxidative stress events of the neurotoxicity caused by paraquat (PQ) has been investigated due to its known antioxidant properties. PQ is a potential environmental neurotoxin related to an enhanced risk for neurodegenerative pathologies and it acts mainly on dopaminergic neurons, which are very sensitive to oxidative stress. The results from the study suggest that the dietary supplement of Na_2_SeO_3_ had a protective effect on the biochemical and behavioral functions of the zebrafish exposed to this neurotoxin [96].

On the other hand, 3-nitropropionic acid (3-NP) is a mitochondrial toxin that is used in experimental models of HD to study some aspects of the physiopathology of the disease, particularly oxidative stress and mitochondrial dysfunction. Although further studies are required, it is believed that the mitogen-activated protein kinases (MAPK) pathways, which can be induced or activated by ROS, are involved in the 3-NP-mediated neurodegeneration. The rise in interest in studying the role of Se in different neurodegenerative processes relies on the fact that this element is involved in the maintenance of correct redox signaling of the biological systems. In view of the former, the beneficial therapeutic effects of Na_2_SeO_3_ have been investigated towards the oxidative stress caused by 3-NP in cortical neurons of mice. The beneficial role of this compound has been well demonstrated and the study provides insights into its mechanism of action. This is related to an increase in the GPx activity that is known to exert a crucial antioxidant role in the brain [97].

AD was described in 1906 for the first time. Even though it is one of the most prevalent neurodegenerative disorders, its etiology is still not well-documented. The major pathological features of AD include the presence of hyperphosphorylated tau-containing neurofibrillary tangles (NFTs)—which result from the aggregation of pyramidal neurons with the tau protein (τ)–, the production of extracellular senile amyloid β (Aβ)-containing plaques, and it is characterized by a progressive loss of cognition [5,98]. At present, the available treatments are mildly effective in the maintenance of cognitive function and are mainly focused on targeting Aβ. It should be pointed out that the brain is particularly reliant on antioxidant mechanisms due to the abundance of oxidizable metals on it and the high oxygen consumption [5]. Given the prominent antioxidant properties of Se, its derivatives have been proposed as potential agents for the treatment and/or the prevention of AD [5,99]. Furthermore, there is emerging evidence that selenoproteins may play an important role in preventing neurodegeneration [98,100,101,102,103]. In this regard, Na_2_SeO_3_ has proven to attenuate amyloid production by reducing the γ-secretase activity [5].

**Figure 3:**
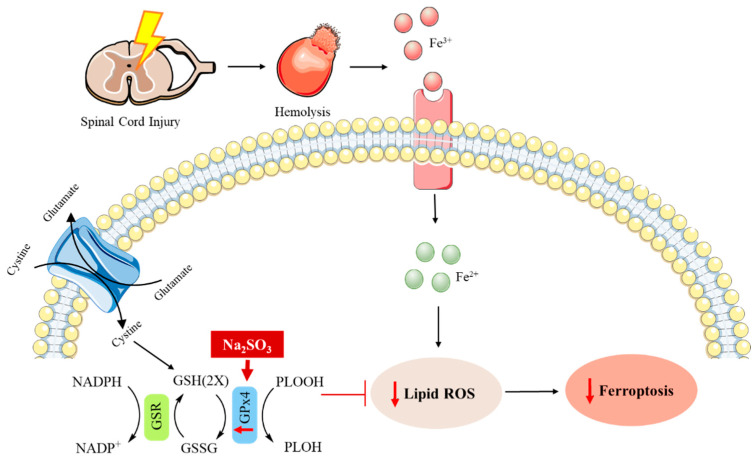
The possible underlying mechanism by which Na_2_SO_3_ boosts the recovery of the neurological function of rats with spinal cord injury through GPx4 [107].

It is worth mentioning that the role of Na_2_SeO_3_ in ferroptosis has also been studied. This is an iron-dependent cell death involved in the pathology of numerous neurodegenerative pathologies, such as autism spectrum disorder (ASD) and spinal cord injury (SCI) [104,105,106,107,108,109,110,111]. ASD is complex neuropathology that involves a great number of heterogeneous neurodevelopmental impairments [112,113]. Despite the increased prevalence of ASD worldwide, few efficacious drugs are available for the treatment of this disease [109]. Bearing in mind that ferroptosis is associated with oxidative stress and neuroinflammation, Se supplementation has been suggested as a feasible approach to ameliorate autism-like behaviors [109]. Furthermore, it should be mentioned that the Se-dependent glutathione peroxidase 4 (GPx4) is a selenoprotein involved in the regulation of this process [114,115]. In this context, the potential application of Na_2_SeO_3_ has been recently studied. It has been demonstrated that this compound can reduce ferroptosis through the depletion of the ROS levels through the Nrf2/GPx4 signaling pathway, which is to say that Na_2_SeO_3_ promotes the expression of GPx4 by the regulation of the nuclear factor erythroid 2-related factor 2 (Nrf2), which is a transcriptional factor involved in the regulation of the oxidative stress response in cells. As a result, the hippocampal damage and abnormal ASD-like behaviors were ameliorated in a mouse model [109]. Likewise, one recent report has shown the protective role of this selenocompound in SCI. This central nervous system injury represents a growing problem for public health owing to its high mortality and morbidity rates, as well as the functional impairments produced in the patient’s body. Nevertheless, there are slightly more efficient available treatments and recovery strategies for this kind of injury [106,116]. The pathophysiology of SCI consists of two phases. The first injury is related to the compression or contusion of the spinal cord produced by mechanical forces, whereas the second phase is associated with the maintenance and development of the first one. Some hallmarks linked to this second injury are oxidative stress, inflammatory reactions, and necrosis or apoptosis, which can result in a decreased functionality of neurons [107,117,118,119,120,121]. In the past few years, several studies have demonstrated that ferroptosis is involved in this second phase of SCI and its inhibition represents an attractive target for the treatment of this injury [111,121,122,123,124,125,126]. As Se had been previously studied in SCI [127,128,129,130,131,132], the role of Na_2_SeO_3_ in ameliorating the neuronal function through the inhibition of ferroptosis has been analyzed. Na_2_SeO_3_ administration in a rat model resulted in an enhancement of the expression and activity of GPx4, thus inhibiting ferroptosis and improving the neurological recovery after SCI [107] (Figure 3).

#### 2.1.7. Sodium Selenite and Viral Infections

A great variety of studies have reported the role of Na_2_SeO_3_ in viral diseases of different etiologies. The Na_2_SeO_3_ treatment in human hepatoma cell lines displayed suppression of the hepatitis B (HBV) protein synthesis and transcription as well as viral genome replication [133]. Additionally, it has proven to be able to promote the proliferation of the natural killer cells and the suppression of the thioredoxin reductases (TrxR) activity, resulting in a decrease in the infectivity of some RNA viruses, such as the Ebola virus [64]. It is important to highlight that TrxR is the main enzyme involved in DNA replication, redox signaling, and defense against the oxidative damage caused by oxygen metabolism [42].

At the present level of knowledge, it has been suggested that the etiology of Keshan’s disease could be related to Se deficiency as well as infection with Coxsackie B virus (CVB) [24,134,135,136,137]. In this regard, several studies showed that the supplementation of Na_2_SeO_3_ in humans can prevent this disease and it has been eradicated from endemic areas [84,135,136,138]. Furthermore, Se levels have been linked to the potential evolution of these viruses to become more virulent. A deficiency of this trace element and reduced GPx1 protective activity can lead to more detrimental symptoms, increased pathogenicity, and the development of myocarditis in the host [5,135,136].

On the other hand, influenza viruses exert a high heterogenicity and can cause seasonal epidemics worldwide, resulting in elevated rates of morbidity and mortality [139,140,141]. They can be classified into four types according to their antigenicity: A, B, C, and D. Among them, influenza A viruses can be considered the most pathogenic for humans [142]. At present, three strains of influenza viruses have led to human pandemics: H1N1, H2N2, and H3N2 [143,144]. Even though great efforts have been made to treat these infections, new antiviral drugs are needed due to the increasing resistance of the influenza viruses to current treatments [144,145]. Recently, the protective role of Na_2_SeO_3_ in the H1N1 influenza virus has been studied in vitro using an MDCK cell model. In these cells, an increase in ROS can be observed [144]. It is a well-known fact that Na_2_SeO_3_ can reduce the production of these species and is related to the induction of apoptosis in the infected cells [42,144]. According to data, the molecular mechanism of its activity relies on the AKT, p53, and MAPK pathways [144]. Therefore, it can be concluded that the use of Na_2_SeO_3_ could be a feasible approach for the development of new antiviral treatment modalities for influenza viruses.

In the past few years, the severe acute respiratory syndrome (SARS) caused by the coronavirus SARS-CoV-2 has afflicted millions of lives. In the most severe cases, it can cause systematic inflammatory responses, myocarditis, and acute respiratory distress [24,42,136]. It is a single-stranded RNA virus that targets cells expressing the angiotensin-converting enzyme 2 (ACE2) entry receptor in the host (i.e., alveolar and airway epithelial cells, vascular endothelial cells, macrophages in the lungs, myocardial and kidney cells) [24,137,146]. Furthermore, it is important to highlight that it can be compared to some other RNA viruses, such as the Coxsackie virus, hemorrhagic viruses (i.e., Ebola virus or Hantavirus), human immunodeficiency virus (HIV), and influenza virus [42]. Although vaccines and drugs have improved a patient’s response to SARS-CoV-2 infection, new approaches for the treatment of this disease are of great interest [147]. In this regard, nutritional strategies have been slowly beginning to emerge. They are believed to ameliorate the long-term complications of this disease, as well as maintain a healthy immune system and reduce the susceptibility of the virus, particularly in places where the treatments for COVID-19 are more limited [136,147]. Some literature reports have indicated that Se, a micronutrient, can reduce the occurrence and severity of several viral infections. This is mainly related to its incorporation into selenocysteine (SeCys), which is a pivotal component of the selenoproteins. These are involved in the maintenance of the redox homeostasis, regulation of the inflammatory cascade, and the modulation of the immune response [135,147,148,149,150]. Furthermore, it should be emphasized that oxidative stress is one of the main features of viral infections (especially respiratory ones) [134,135,136,147]. Keeping in mind the aforesaid antecedents, Na_2_SeO_3_ could be considered an attractive potential adjuvant in the treatment of COVID-19 due to its capacity to reduce oxidative stress and restore the activity of some Se-enzymes. It is also able to tackle some cardiovascular disorders, such as coagulopathy, microthrombotic events, and endotheliitis [42]. Moreover, Na_2_SeO_3_ can prevent the thiol/disulfide exchange started by the protein disulfide isomerases. The role of these redox enzymes is to regulate the thiol/disulfide balance in the interaction between ACE2 and the spike proteins of the SARS-CoV-2/CoV-2 in the host cell membranes, thus blocking the capacity of this virus to cross the cell membrane and enter the healthy cells [5,7,24,137,149].

## 3. Methylselenol Precursors

### 3.1. Methylseleninic Acid

Methylseleninic acid (MSA, MSeA, CH_3_SeO_2_H) is a monomethylated organoselenium compound, part of the group of oxoacids that can be obtained by the oxidative decomposition of methylselenocysteine [13,65]. It can be directly metabolized to methylselenol (CH_3_Se^−^) through different reducing agents by simple non-enzymatic and enzymatic reactions [24,36]. Moreover, it is important to highlight that glutathione (GSH) and NADPH play an important role in these reactions [23,36,151,152]. In the course of the studies, it has been found that MSA exhibits multiple beneficial therapeutic properties, some of which are briefly described in this review.

#### 3.1.1. Methylseleninic Acid and Cancer

When studying the effect of MSA on cancer cells, it was found that it can exert chemopreventive and anticarcinogenic activities. This has been demonstrated in several in vitro and in vivo experiments on a wide range of cell lines, such as prostate, head and neck, leukemia, breast, colon, liver, lung, ovarian, hepatic, pancreatic, and esophageal squamous cancer cells [13,62,63,151,153,154,155,156,157,158,159,160,161]. Nevertheless, a better understanding of the underlying mechanism of action is still required as it remains sparse. At the present level of knowledge, MSA has been demonstrated to display an antiangiogenic activity, arrest the cell cycle in the G1 phase and inhibit the proliferation of several tumoral cells through diverse mechanisms that induce apoptosis [62,63,151,158,159,160,162,163]. Furthermore, MSA can enhance the generation of ROS as well as decrease the intracellular levels of glutathione, resulting in a more oxidized environment that can lead to cell death [65,151]. There is also emerging evidence that MSA can be involved in entosis. This is a programmed cell death that occurs in cancer cells in which one epithelial cell is internalized by another and thereupon it is killed and digested [164,165,166]. It has been demonstrated that MSA can induce entosis in pancreatic cancer cells by cell detachment via the downregulation of two important factors: the cell division control protein 42 homolog (CDC42) and its downstream effector integrin-β1(ITGB1, CD29) [165]. On the other hand, it has been well-documented that MSA can sensitize cancer cells to radiation and several chemotherapeutic drugs, i.e., cisplatin, paclitaxel, etoposide, and doxorubicin [23,65,154,157,167]. Bearing in mind the abovementioned antecedents, MSA can be considered a promising and viable candidate for the treatment of cancer.

#### 3.1.2. Methylseleninic Acid and Endometritis

At present, antibiotic therapy is not enough to treat endometritis. This is a reproductive endocrine disorder and a chronic inflammatory disease that can lead to a decrease in fertility and productivity due to a disruption of the hormonal balance [46,168,169]. *S. aureus* is the main causative agent of this disease as well as other invasive infections. Even though it is not considered a significant intracellular pathogen, it should be pointed out that it can induce apoptosis by invading epithelial and endothelial cells. Moreover, it can produce damage to the uterus, increase the levels of some pro-inflammatory cytokines (i.e., TNF-α and IL-1β) and reduce the tight junction protein expression in the uterus tissue [46]. Several studies indicate that *S. aureus* can be recognized by Toll-like receptor 2 (TLR2) and other TLR family members that are part of the innate immune system [24,46,170].

It is a well-known fact that a large number of selenocompounds exert anti-inflammatory effects [13,24]. For this reason, the protective activity of the MSA was evaluated against *S. aureus*. It was demonstrated that this selenoderivative was able to reduce the expression of the caspase pathways proteins which are a family of cysteine acid proteases involved in the regulation of apoptosis. Thus, the TLR2-related inflammation signaling pathway was attenuated with a depletion of the phosphorylated NF-κB pathway proteins [170]. Therefore, it can be considered that MSA is able to protect against the inflammatory lesions caused by *S. aureus* in the rat uterus. Furthermore, the study showed a decrease in the levels of TNF-α and IL-6 (inflammatory cytokines) which confirms that Se can prevent the invasion of the uterus by this pathogen [170]. Bearing in mind the aforementioned facts, MSA supplementation can be considered a potential strategy for the prevention, control, and treatment of *S. aureus*-induced endometritis.

#### 3.1.3. Methylseleninic Acid and COVID-19

Over the last three years, several studies focusing on the relationship between Se and SARS-CoV-2 have been reported [5,24,42,64,136,137,147,148,149,171]. There is a growing body of evidence that the cysteine 145 residues of SARS-CoV-2 M membrane glycoprotein plays a key role in the replication of this virus [5,137,148]. For this reason, it is considered an excellent target for reducing the replication, transcription, and truncating of the life cycle of the SARS-CoV-2 virus.

Many studies have documented that MSA has a strong redox activity that allows it to modify any proteins that contain thiols in their structure [36,148,172,173]. For this reason, it was hypothesized that this redox-active Se compound could react with the HS-Cys145-M^pro^, thus blocking the virus’s capacity to multiplicate [137,148]. Another fact that should be considered is that in viral respiratory infections there is enhanced ROS production, increased oxidative stress, and higher levels of oxidation products in the infected cells [13,135,148,150]. Contrary to what happens in normal cells, it is known that in this case methylselenol is converted into MSA and is retained in the infected ones [137,148]. The accumulation of MSA might modify the Cys145 residue, resulting in the inactivation of the M^pro^ of SARS-CoV-2, but further studies would be required to prove that.

#### 3.1.4. Methylseleninic Acid and *Mycobacterium tuberculosis* Infection

*Mycobacterium tuberculosis* (MTB) is a bacterium that can cause pulmonary tuberculosis (PTB) which is a major health threat worldwide. It is estimated that about 10 million people were affected by it in 2020, according to data provided by the WHO [174]. The interest in finding new anti-MTB agents has been raised due to the increasing drug resistance to the treatments available [175,176,177,178]. Recent studies have focused their attention on the regulation of the immune system of the host by different nutritional supplementation as it is considered a safe and effective alternative [179,180]. It should be outlined that several studies have focused their attention on exploring the potential application of Se as an anti-MTB agent [179,180,181,182]. Moreover, there is growing evidence that there is a relationship between Se levels and the development of PTB [179,183]. Bearing this in mind, the effect of MSA to counteract this infection has been studied. There is ample evidence that this organic Se compound exerts an antimicrobial activity through the activation of the c-Jun-mediated autophagy and LC3-associated phagocytosis (LAP) of alveolar macrophages infected with MTB, thus limiting its intracellular growth [24,179]. These findings should be taken into consideration in the future to provide a new anti-MTB therapy based on an appropriate Se supplementation.

### 3.2. Se-methylselenocysteine

*Se*-methylselenocysteine (MSC, MeSeCys, C_4_H_9_NO_2_Se) is a natural monomethylated selenoamino acid and one of the most well-studied selenocompounds. It can be found in plants with high content of Se, such as garlic and broccoli florets [62,137,184,185]. It results from the methylation of SeCys and is an analog of *S*-methylcysteine. It should be noted that MSC is chemically less reactive than many Se organic derivatives [24,186]. Notably, it has been proposed as a nutritional supplement candidate because it is a relatively stable free amino acid, is not immediately incorporated into proteins, and can be accumulated after ingestion [24,187]. This compound is metabolized into β-methylselenopyruvate (MSP) or monomethylselenol by the kynurenine aminotransferase (KYAT1, CCBL1) [24,188]. Several studies have unveiled that this Se compound displays several properties that could play an important role in some pathologies. Herein, we present a survey of the literature that demonstrates the diverse possible therapeutic applications of MSC.

#### 3.2.1. *Se*-methylselenocysteine and Alzheimer’s Disease

AD is a chronic, irreversible and devastating neurodegenerative disease that affects millions of people worldwide and is one of the main reasons behind dementia in the elderly. One of the main features of AD is the abnormal aggregation of the proteins Aβ and τ. This is associated with synaptic loss, memory decline, and neuronal dysfunction. Moreover, both proteins can induce oxidative stress by the production of ROS and dysregulation of the homeostasis of some metal ions which are involved in the development of AD [184,189].

Research activities on MSC in the AD are sparse. Nevertheless, recent studies have suggested that this compound could ameliorate neuropathology and cognitive deficits through the attenuation of oxidative stress and metal dyshomeostasis (copper especially) in the triple-transgenic (3 × Tg-AD) mouse model of AD [184]. This results in the suppression of the abnormal activation of the MEK/ERK pathway, probably due to its metal-chelation and antioxidant properties, thus preserving the normal function of neurons as well as the memory and learning skills of the AD mice [189]. Furthermore, it has been shown that the treatment with MSC is a feasible approach for this disorder as it can modulate the expression of mitochondrial-related proteins, thus reversing AD by improving the pathological symptoms and changes observed in AD mice [184].

#### 3.2.2. *Se*-methylselenocysteine and Cancer

Many organic selenoderivatives have been considered suitable agents in cancer treatment. This is based on the fact that, in comparison with inorganic Se compounds, they can cross the cell membranes more efficiently, produce fewer side effects and exhibit lower systemic toxicity [190]. There is emerging evidence that MSC can alter several signaling pathways, thus strikingly exerting tumor-specific cytotoxic activity. Although it is not considered an active drug, it has been well-established that its metabolites, such as methylselenol and MSP, display antitumoral and antiproliferative properties [188]. The significant role of methylselenol in antitumoral treatment lies in its capacity to induce cell death and it is recognized as one of the most cytotoxic Se compounds [23,72,188,190,191]. Likewise, MSP also exhibits interesting properties, such as its ability to inhibit angiogenesis and histone deacetylase (HDAC) [72,192,193]. The cytotoxic activity of MSC has been determined in a wide range of in vitro studies in different human cancer cell lines, such as breast, esophagus, liver, lung, ovarian, and head and neck squamous cells [63,72,158,190,192,194,195]. Furthermore, it has been shown to deplete the levels of the vascular endothelial growth factor (VEGF) and the hypoxia-inducible factor-1-α (HIF1-α) in various cell cultures and animal models [63,193,196,197]. Regarding its pertinent mechanism of action, it has been suggested that MSC is associated with caspase-dependent apoptosis, albeit further studies are required [34,63,193].

On the other hand, several in vivo studies have been carried out to evaluate the anticancer potential of MSC. They are mainly based on the combination therapy of this compound with some of the available chemotherapeutic drugs to improve their efficacy [63,198]. In fact, MSC has attracted prominent attention not only for its capacity to act synergistically with a great number of compounds used in cancer treatment but also for its role in the modulation of some of the processes involved in metastasis [63,190,197]. Beyond this, several studies on animal models have reported that it exerts protective effects against the toxicity associated with chemotherapeutic drugs [63]. Bearing in mind the aforesaid antecedents, the great potential of MSC as a chemotherapeutic agent should be considered.

#### 3.2.3. *Se*-methylselenocysteine and Vulvar Candidiasis

Vulvar candidiasis (VVC) is a vaginal mucosa infection mainly caused by *Candida* species, especially *Candida albicans* (*C. albicans*). Notably, it has been reported that it is experienced by a great number of women (75–80%) at least once in their lifetime and it can have an impact on the quality of their lives [199,200,201,202]. This is a major ongoing clinical issue due to the increased resistance to the available treatments for this infection [201,202,203,204]. Thereby, discovering new therapeutic alternatives and strategies is of great importance. In this regard, the potential application of MSC has been recently studied. It was found that this selenocompound can inhibit the growth of the pathogen in a concentration-dependent manner. In addition, to elongate the time that MSC remains in the vagina and improve its effect, it was loaded in a mucoadhesive thermogel (NAC-HA thermogel) resulting in the L-SeMC@NAC-HA thermogel, which showed a sustained release profile in vitro and was able to enhance the retention time of MSC in the vagina tract. Moreover, in vivo studies demonstrated that it has a good safety profile and can reduce the number of *C. albicans* present in the vaginal secretions, ameliorate the damage produced by this commensal fungus and diminish some pro-inflammatory factors (TNF-α, IL-1α, and IL-β) [205]. In view of the former, this thermogel could be considered a promising agent for the treatment of VVC.

#### 3.2.4. *Se*-methylselenocysteine and Ischemic Stroke

Since 2012, ferroptosis has attracted scientists’ research interest as it is related to several brain pathologies [206,207,208,209]. At the present level of knowledge, it is known that this non-apoptotic programmed cell death pathway is associated with cell death due to a rise in the levels of lethal lipid hydroperoxides which causes a disturbance in the membrane integrity [111,210]. Moreover, GPx4, a selenoprotein present in the brain tissue, has been reported as a pivotal protein involved in the regulation of ferroptosis. Notably, Se has proven to increase the levels of this protein, thus enhancing its antioxidant properties [107,114,115]. Likewise, it has been reported that it can enhance other anti-ferroptotic genes. It has been demonstrated that these facts can lead to the protection of neurons and prevent hemorrhagic and ischemic stroke [114,211]. Evaluation of MSC in an in vitro ischemic stroke model, showed it to possess anti-ferroptotic properties. Owing to its favorable toxicity profile in a preclinical animal model, MSC was also studied in vivo in MCAO model mice, demonstrating a reduction in the infarct volume and the neurological deficits related to cerebral ischemia. Due to the abovementioned reasons, MSC can be suggested as a ferroptosis inhibitor that has the potential to prevent neuronal damage and death caused by ischemic stroke [114]. Furthermore, it should be noted that this compound could be used in other neurological disorders where ferroptosis is involved (i.e., Parkinson’s disease and Alzheimer’s disease) [114].

#### 3.2.5. *Se*-methylselenocysteine and Wound Healing

Wound healing has drawn widespread attention from the scientific community as there is an increased interest in discovering new ways to accelerate the healing process and elude the formation of scars [212]. It should be highlighted that it is a complex biochemical and cellular process that involves different phases. Cell migration is considered to be the most limiting step in the process, and it is necessary for the re-epithelialization that occurs in the proliferation phase [213,214]. Keratinocytes are the principal component of the epidermis and play a crucial role in the re-epithelialization [213,215]. At the wound site, they undergo the epithelial-mesenchymal transition (EMT) process which is known to produce migratory mesenchymal cells from adherent cells [213,214]. Some literature reports have indicated that the loss of E-cadherin and the nuclear accumulation of β-catenin are required factors for this process. Moreover, matrix metalloproteinases (MMPs) are involved in different essential healing processes, such as EMT, cell migration, and remodeling the granulation tissue [216].

A large body of existing literature indicates that redox-regulated processes play a key role in wound healing, and it is a well-known fact that a wide range of selenocompounds exert antioxidant activity [67,217,218]. It should be outlined that MSC has been associated with the reinforcement of the antioxidant capacity of cells, thus protecting them from oxidative stress and inhibiting apoptosis [187,188,213,219]. Currently, little data are available about the effect of MSC on wound healing. Nonetheless, a recent study has investigated its effects on EMT and the possible underlying mechanism by using HaCaT keratinocytes. This compound displayed a protective effect on the cells from excessive oxidative stress by activating the antioxidant response. Furthermore, it enhanced the keratinocyte migration, which leads to skin wound healing through the stimulation of EMT by the β-catenin pathway. This was evidenced by a shrinkage in the levels of E-cadherin in the cells present in the wound edge and increased levels of MMPs and some key transcription factors (Snail and Twist) involved in the induction of this process. In addition, MSC prevented the degradation of β-catenin, resulting in its accumulation in the nucleus and the activation of the β-catenin signaling pathway [213].

### 3.3. Selenomethionine

Selenomethionine (SLM, SeMet, C_5_H_11_NO_2_Se) is a selenated monomethylated methionine analog and is the main form of Se in foods such as rice, cereals, beans, and nuts [220,221,222]. It is produced in fungi and plants and can replace methionine in different enzymes and proteins, thus endowing them with a higher redox activity [220]. Amongst the range of Se compounds, SeMet is one of the most easily absorbed by the human body and is considered a pivotal precursor of methylselenol [1,223,224]. Once it is absorbed, it can either be accumulated in proteins or metabolized into reactive Se forms [24]. Over decades, an extensive number of publications have reported the beneficial therapeutic effects of SeMet in a great variety of pathologies. Herein, we aim to provide an overview of the latest data available about it.

#### 3.3.1. Selenomethionine and Autoimmune Thyroid Diseases

In the past few years, a major endeavor has been directed towards the research of new treatments for autoimmune thyroid diseases (AITDs). Its incidence has markedly increased over the years, and it is mainly diagnosed in middle-aged women. AITDs encompass a wide spectrum of disorders. Amidst them, Graves’ disease (GD) and Hashimoto’s thyroiditis (HT) are the most common ones [225,226,227,228,229].

Se is an essential micronutrient that exerts a critical role in the maintenance of several cellular functions. It is a well-known fact that the thyroid organ is particularly rich in Se, which is incorporated into selenoproteins, such as glutathione peroxidase (GPx), thioredoxin reductase (TrxR), iodothyronine deiodinases (DIO), and selenoprotein P (SELENOP). Some of them are involved in hormone metabolism and have a crucial antioxidant activity directed against the oxygen free radicals generated in the production of the thyroid hormones (THs) [225,226,228,230,231,232]. Furthermore, there is mounting evidence that patients with thyroid pathologies (i.e., hypothyroidism, AITDs, and enlarged thyroid) present reduced levels of this micronutrient [225,233]. Furthermore, it should be highlighted that there is a linking bond between AITDs and environmental factors that can trigger oxidative stress which is considered a key pathogenic hallmark in AITDs development [234,235,236]. For this reason, the antioxidant properties associated with Se have drawn great attention [237]. Thus, a wide range of trials has been conducted to determine whether Se supplementation could influence the evolution of these diseases [225,236,238,239,240].

In this context, a large variety of studies have been carried out to ascertain the role of SeMet on AITDs. Over the last years, several literature reports have indicated that this element, in combination with myo-inositol, has a beneficial effect as it can reduce the thyroid-stimulating hormone (TSH) and the antithyroid autoantibodies levels, thus decreasing the risk of progression to hypothyroidism in AITDs patients [225,237,241,242,243,244,245]. Moreover, the protective effect of Se supplementation on thyroid autoimmunity during and after pregnancy has been studied [5,228,238,239]. In this regard, the SERENA study has evaluated the effect of SeMet on AITDs in euthyroid pregnant women with positive antithyroid antibodies. The results indicate that giving 83 μg/day of SeMet has a beneficial outcome as it reduces the autobody titer during pregnancy and the recurrence of postpartum thyroiditis [246,247]. Furthermore, a review paper has revealed that 200 µg/day could reduce thyroid inflammatory activity, the risk of postpartum thyroid dysfunction, and the development of permanent hypothyroidism [226]. In view of the former, further research is required to assess and clarify the efficacy and safety of Se supplementation in pregnancy to avoid adverse events. On the other hand, the effect of SeMet on the evolution of HT has been addressed in multiple publications, but its exact benefit and underlying immunological mechanism remain unclear [5,226,248,249,250]. Data obtained from the SETI study indicate that short-term supplementation with SeMet resulted in a normalization of the TSH levels in 50% of the patients that presented subclinical hypothyroidism related to chronic AITDs. Moreover, it evaluated the capacity to modulate the secretion of interferon-induced chemokines (CXCL9, CXCL10, and CXCL11), which are responsible for the progression of diverse autoimmune diseases. Results showed that the SeMet supplementation reduced the levels of these chemokines [24,251]. In addition, research has been carried out to determine the effect of SeMet and levothyroxine (LT4) on the systemic inflammation and release of cytokines in HT patients. Despite affecting different inflammatory cells, they displayed similar anti-inflammatory effects, which resulted in a depletion of the antibody titers to thyroid peroxidase. This is an interesting fact as it might suppose a clinical advantage in the treatment and prevention of this disease by using two agents at once [24]. Furthermore, in mild Graves’ orbitopathy, an autoimmune inflammatory disease that can be founded in Grave’s patients, the use of SeMet (100 g/day) is recommended as it is associated with a decrease in the progression of this disease as well as an improvement of life quality and the ophthalmic outcomes [226]. This has been included in the European Group on Graves’ Orbitopathy 2021 guidelines [252]. Based on the above, these insights could aid future investigations aimed at discovering and improving therapeutic strategies for the treatment of AITDs.

#### 3.3.2. Selenomethionine and Cancer

Experimental data of recent years devoted to the role of SeMet in cancer treatment have shown that it has a notable cytotoxic activity in numerous cancer cell lines, i.e., breast, colorectal, prostate, melanoma, and cholangiocarcinoma [63,150,173,220,253,254,255]. It is noteworthy that this antitumoral effect has been achieved at a higher concentration than the ones observed for other common redox-active Se derivatives. Nonetheless, it has shown a great selectivity towards cancer cells over normal ones [63]. It should also be noted that the exact mechanism of action of SeMet in cancer remains unclear. In this regard, several pathways have been suggested to be involved in the SeMet-induced apoptosis, such as HDAC inhibition, the activation of caspases and p53, decreased cyclooxygenase-2 expression, reduced GxP activity, endoplasmic reticulum stress, and altered Bcl-xL, Bax, Bad and Bim expression [63,253]. Furthermore, a comparative study of the safety and pharmacokinetic (PK) profile of SeMet, MSC, and Na_2_SeO_3_ in cancer patients has shown that greater systemic exposure occurs with SeMet. This could be related to the fact that this selenocompound can be incorporated into proteins in place of methionine. As a result, higher systemic levels are accumulated and there is a delayed total body clearance. It should be highlighted that these proteins containing SeMet can act as redox-active compounds and exert the anticancer activities associated with this selenoderivative [61,220]. Bearing in mind the aforesaid antecedents, the involvement of Se in cancer treatment can be considered a suitable therapeutic alternative.

#### 3.3.3. Selenomethionine and Cardiovascular Diseases

Accumulating evidence indicates that oxidative stress is a prominent feature of CVD [256,257,258,259]. Thereof, antioxidants administration has raised interest in the past years owing to their capacity to act as potential scavengers of free radical species [260,261,262]. Since Se species are able to react with a great variety of oxidants, such as hydrogen peroxide (H_2_O_2_) and hypochlorous acid (HOCl) and promote the activity of endogenous Se-dependent antioxidants (i.e., GPx and TrxR systems), they have been proposed as an alternative therapeutic strategy to counteract oxidative stress [263,264]. Amongst all the Se compounds, SeMet has been proposed as a suitable candidate because it is immediately taken up by the cells incorporated into the cellular proteins instead of the methionine and endows them with extra redox properties [220,260]. It has been shown that SeMet can react readily with HOCl to produce the corresponding selenoxide which can be catalytically recycled, thereby reducing the damage produced by HOCl [60,263,265]. For instance, it can reduce the damage induced by HOCl in VSMCs, which are involved in the development and progression of atherosclerosis [263,266]. Moreover, SeMet supplementation has been reported to improve the vessel’s function, render a stable lesion phenotype, decrease the inflammatory macrophages present in the lesion and modify the acute inflammatory response [266]. In addition, a study of the role of SeMet in peripartum cardiomyopathy (PPMC) patients with left ventricular systolic dysfunction and Se deficiency has been recently published. PPMC is non-ischemic cardiomyopathy associated with signs and symptoms of heart failure and high rates of thromboembolic events [267,268,269]. Although Se was not able to diminish the risk of the primary outcome, SeMet significantly decreased the symptoms related to heart failure and a reduction in the all-cause mortality tendency was observed [82,267].

#### 3.3.4. Selenomethionine and Intestinal Ischemia-Reperfusion Injury

Ischemia-reperfusion (I/R) injury is a manifestation of organ or tissue damage and is caused by an initial interruption of the blood flow in them followed by its subsequent restoration [270,271]. It has been thoroughly investigated in several diseases (i.e., cardiac, hepatic, and cerebral pathologies) as well as intestinal transplantation and injury. Among all, the intestine has been demonstrated to be one of the most sensitive to it [270,272]. Inflammation and intestinal mucosal apoptosis are hallmarks related to intestinal I/R. Moreover, cell dysfunction caused by this injury contributes to cellular damage that can lead to structural changes, cell metabolism disorder, necrosis, and apoptosis [273,274,275]. Over the last few years, the role of SeMet on I/R has been studied. Although it has proven to alleviate this disorder, few data are available about its mechanism of action. Results from a recent study suggest that it can inhibit apoptosis related to I/R by increasing its antioxidant capacity, thus maintaining the redox homeostasis. Additionally, it can repress mitochondrial dysfunction through the inhibition of caspase-3 and the expression of two apoptotic proteins (Bax and cytochrome c), along with an enhancement of the Bcl-2 apoptotic protein levels [273]. Therefore, it can be concluded that SeMet could be a valuable therapeutic approach to preventing I/R damage.

#### 3.3.5. Selenomethionine and Kidney Diseases

Ochratoxin A (OTA) is a mycotoxin produced by *Aspergillus* and *Penicillium* species and can mainly induce nephrotoxicity in humans exposed to OTA-contaminated food, which can have a great impact on health [276,277,278]. Moreover, it has been hypothesized that it could have a likely relationship with Balkan nephropathy [278,279]. It is a well-known fact that OTA can induce nephrotoxicity through oxidative stress and apoptosis, as well as activating autophagy and inhibiting the synthesis of proteins [280]. There is emerging evidence that supports the notion that supplementation with melatonin and zinc can protect from mycotoxin contamination [281,282]. Additionally, there have been a few reports showing that SeMet supplementation can reduce the intracellular ROS levels, thus mitigating the OTA-induced apoptosis and immunotoxicity [283]. In this respect, the effect of the combination of zinc and Se on OTA-induced fibrosis in the human renal proximal tubule epithelial (HK-2) cell line has been studied. According to data, both exerted a synergistic protective activity, which was associated with the inhibition of the ROS-dependent autophagy in these cells [284]. Moreover, the SeMet protective effect on the cytotoxicity and pyroptosis of Madin–Darby canine kidney (MDCK) cells induced by OTA has been demonstrated. Indeed, it has been able to decrease the ROS levels and inhibit the activation of the NLRP3 inflammasome and caspase-1 pyroptosis in MDCK cells [280].

In the same manner, the joint activities of SeMet and icariin (ICA) were assessed in chronic tubulointerstitial nephritis (CTIN) as it is one of the most common kidney diseases. It has been proven that this combination displays a nephroprotective effect as it can reduce renal dysfunction, cell apoptosis, pathological damage, and tubulointerstitial fibrosis in a CTIN mouse model and HK-2 cells. Moreover, it should be highlighted that the combination showed a better effect than the single agent ICA or SeMet. Furthermore, the underlying mechanism may be attributed to blocking the TLR4/NFκB signaling pathway [285].

#### 3.3.6. Selenomethionine and Neurodegenerative Pathologies

**Figure 4:**
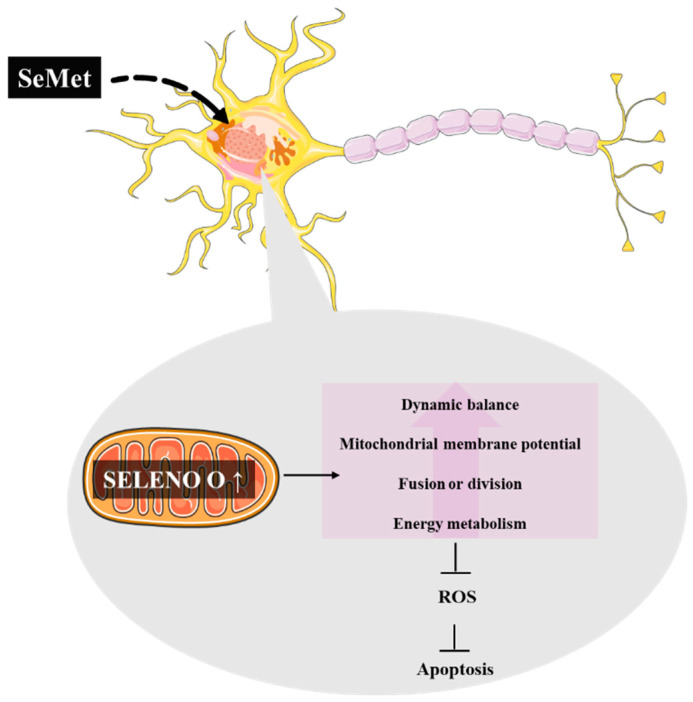
SeMet can improve the mitochondrial function in a model of AD through the upregulation of SELENO O, which is present in the mitochondria [290].

Among the diverse health benefits ascribed to SeMet, its role in AD has received great attention. Several studies in triple transgenic (3 × Tg)-AD mouse models have unveiled its capacity to ameliorate cognitive impairments, as well as decrease synaptic damage and inhibit the amyloid plaques and neurofibrillary tangles generation [286,287,288,289,290]. Thus, it has exerted a significant role in the protection of neurons and maintenance of the nervous system functionality in a particular (3 × Tg)-AD mouse model [290]. In this in vivo study, it has been reported that the treatment with SeMet resulted in a rise in the number of mitochondria, as well as the mitochondrial membrane potential, where a significant reduction in the levels of ROS and apoptosis was observed. Noteworthy is an enhancement of the mitochondrial selenoprotein O (SELENO O) expression resulted from the SeMet treatment [290]. This upregulation of SELENO O in AD cells is interesting as Se mainly displays its effects through selenoproteins and it opens up new perspectives in the search and development of new drugs for AD (Figure 4).

On the other hand, some literature reports have indicated that neurogenesis plays a pivotal role in the preservation of the neural system function and its induction is considered a feasible approach for the treatment of AD [291,292,293,294]. For this reason, the potential application of SeMet to the regulation of hippocampal neurogenesis has been studied in cultured hippocampal neural stem cells (NSCs) from 3 × Tg-AD mice. It has been reported that this Se compound can promote the proliferation of NSCs and increase the number of neurons differentiated, both in vitro and in vivo. As a result, the damaged neuronal systems of AD mice can be repaired. Moreover, it should be noted that SeMet exerts its effects by modulating the PI3K-Akt-GSK3β-Wnt signaling pathway [288]. In view of the former, these findings and their implications should be taken into consideration for further studies to clarify the real multi-faceted therapeutic potential of SeMet on AD.

#### 3.3.7. Selenomethionine and Viral Infections

Due to the COVID-19 outbreak, the topic of the therapeutic role of SeMet against viral infections has raised interest. In vitro studies have demonstrated the antiviral potential of this compound in several single-stranded RNA and DNA viruses, i.e., hepatitis C (HCV), Coxsackie virus, HIV, porcine circovirus type 2 (PCV2), and porcine delta coronavirus (PDCoV) [24,136,220,295,296,297,298]. Nevertheless, future research should be designed by carefully considering the different concentrations used in these studies to highlight the real potential effects of this selenocompound.

On the other hand, it should be noted that one of the components of the Curvic^TM^ (SSV-003) formulation is SeMet. This herbal drug showed in vitro and in vivo efficient antiviral activities against influenza A virus (H1N1) (ATCC^®^ VR-219^TM^) and human beta coronavirus (ATCC^®^ VR1558^TM^). Furthermore, no morbidity or mortality was observed in the acute oral toxicity study [24,299].

In view of the former, SeMet can be considered a suitable therapeutic agent against viral infections owing to its multiple properties as it can act as an antioxidant, along with redox and immune system modulator. In this regard, SeMet has also been proposed as a potential candidate for the treatment of the SARS-CoV-2 infection, although little data are available at the present level of knowledge [24,300].

## 4. Conclusions

Currently, the development of new drugs presents several setbacks, such as toxicity, solubility, and poor pharmacological profiles. In this context, the use of prodrugs is a feasible approach to overcome these issues. Among them, Se compounds that can render the two main Se-metabolites (H_2_Se and CH_3_SeH) have raised great interest in the last decade. The most studied ones are SS, MSA, MSC, and SLM, which have proven to display potent biological activities towards several pathologies, including cancer, viral infections, and neurodegenerative and cardiovascular diseases.

The pieces of evidence gathered in this review point out that the searching for new precursors of these metabolites would be a promising toolkit to be considered in the discovery of safer and more efficient derivatives for treating a plethora of illnesses.
